# Development and validation of an oxidative stress—associated prognostic risk model for melanoma

**DOI:** 10.7717/peerj.11258

**Published:** 2021-04-20

**Authors:** Yu Yang, Xuan Long, Kun Li, Guiyun Li, Xiaohong Yu, Ping Wen, Jun Luo, Xiaobin Tian, Jinmin Zhao

**Affiliations:** 1 Department of Orthopedics Trauma and Hand Surgery, The First Affiliated Hospital of Guangxi Medical University, Nanning, China; 2 Department of Obstetrics and Gynecology, The Affiliated Hospital of Guizhou Medical University, Guiyang, China; 3 The Second People’s Hospital of Guiyang, Guiyang, China; 4 Guizhou Medical University, Guiyang, China

**Keywords:** Skin cutaneous melanoma, Oxidative stress, Prognostic signature, Risk model, Bioinformatics analysis

## Abstract

**Background:**

Oxidative stress (OS) is key to various diseases and is implicated in cancer progression and oncogenesis. However, the potential diagnostic value of OS-related genes in skin cutaneous melanoma (SKCM) remains unclear.

**Methods:**

We used data of RNA sequencing from 471 tumor tissues and one healthy tissue acquired from The Cancer Genome Atlas (TCGA)-SKCM cohort. The Genome Tissue Expression database was used to acquire transcriptome data from 812 healthy samples. OS-related genes that were differentially expressed between SKCM and healthy samples were investigated and 16 prognosis-associated OS genes were identified. The prognostic risk model was built using univariate and Cox multivariate regressions. The prognostic value of the hub genes was validated in the GSE65904 cohort, which included 214 SKCM patients.

**Results:**

The overall survival rate of SKCM patients in the high-risk group was decreased compared to the low-risk group. In both TCGA and GSE65904 cohorts, the ROC curves suggested that our prognostic risk model was more accurate than other clinicopathological characteristics to diagnose SKCM. Moreover, risk score and nomograms associated with the expression of hub genes were developed. These presented reiterated our prognostic risk model. Altogether, this study provides novel insights with regards to the pathogenesis of SKCM. The 16 hub genes identified may help in SKCM prognosis and individualized clinical treatment.

## Introduction

Skin cutaneous melanoma (SKCM) is an aggressive cancer that has been recognized as a relevant cause of death (Ekwueme et al., 2011). Indeed, SKCM is the most frequent cause of death in patients with skin tumors (Holmes, 2014; Liu-Smith, Jia & Zheng, 2017). Early diagnosis and treatment of SKCM are crucial for a favorable prognosis (Hamm et al., 2008); however its pathogenesis remains unclear. Previous studies showed that the degree of skin pigmentation is associated with the progression and occurrence of SKCM (PDQATE Board, 2002; Kanavy & Gerstenblith, 2011). Genetic susceptibility, acquired melanocytic nevi, and family history also play key roles in disease pathogenesis (Gilchrest et al., 1999; Hawkes, Truong & Meyer, 2016). However, it is often difficult to use the above factors to facilitate early diagnosis, making the development of better tools to diagnose early SKCM an important objective in the field (Eisenstein et al., 2018). Therefore, understanding the molecular mechanisms of SKCM and exploiting effective early diagnosis indicators may have a great impact on the survival rate and long-term quality of life of SKCM patients.

The occurrence of oxidative stress (OS) is due to the unbalance between cellular oxidant and antioxidant systems due to various internal and external factors that ultimately lead to the generation of reactive oxygen species (ROS). These are comprised of reactive nonradical species and free radicals, e.g., singlet oxygen, superoxide anion, and hydrogen peroxide (Lü et al., 2010). Excessive ROS can lead to double-stranded DNA breaks and genotoxicity, eventually leading to genomic mutations and tumorigenesis (Moloney & Cotter, 2018; Wang et al., 2017; Zhou, Shen & Claret, 2013). The expression of OS genes plays a crucial role in physiological homeostasis and is associated with the development and progression of several human diseases, such as osteoporosis (Almeida & Porter, 2019), neurodegenerative (Buendia et al., 2016), and inflammatory diseases (Thomson, Hemphill & Jeejeebhoy, 1998). However, both the molecular association between OS genes and SKCM, and their impact on early prognosis, are poorly understood.

Previous studies have described the relationship between OS and its effects on tumorigenesis and disease progression of different tumors (Gill, Piskounova & Morrison, 2016; Klaunig, 2018). For example, in oral squamous cell carcinoma, differential expression of OS-related genes provides a potential basis for clinical drug treatment and clinical decision-making (Pedro et al., 2018). In SKCM patients, the concentration of ROS is reported to be elevated (Liu-Smith, Dellinger & Meyskens, 2014), yet only a few potential mechanisms underlying the roles of OS genes in SKCM have been evaluated. To our knowledge, no systematic study has investigated if OS hub genes are correlated with the prognosis or progression of SKCM. In our study, we obtained the expression profiles of healthy skin and SKCM samples from The Cancer Genome Atlas (TCGA) and the Genome Tissue Expression (GTEx) databases to investigate hub genes related to SKCM prognosis. Subsequently, a prognostic risk model was constructed using the identified OS-related genes and the clinical significance and function of each OS gene in SKCM were systematically explored.

## Materials and Methods

### Processing of raw data

RNA sequencing samples from 472 individuals were obtained from the TCGA database, which comprised of 471 SKCM samples and one healthy skin tissue sample (https://portal.gdc.cancer.gov/). In addition, to increase the number of healthy samples, we collected 812 RNA sequencing data from healthy skin tissues obtained from the GTEx database (https://gtexportal.org/home/datasets) (Human Genomics, 2015; Gentles et al., 2015). A total of 1399 OS-related genes with relevance score ≥ 7 were collected from the Gene Cards database (https://www.genecards.org). Under —log2 fold change—≥ 2 and a standard false discovery rate (FDR) <0.05, an R package was used to detect genes differentially expressed in SKCM and healthy skin samples (Li et al., 2020). Meanwhile, an average count cutoff of 1 was used to eliminate genes. After univariate and multivariate Cox analyses, 16 OS-related genes associated with SKCM prognosis were obtained for the construction of the risk model. We used the National Center for Biotechnology Information-Gene Expression Omnibus database to download the GSE65904 dataset, a cohort of 214 SKCM patients for external verification. The basic characteristics of these SKCM samples were all displayed in Table 1.

**Table 1:** Basic characteristics of the clinical variables in SKCM patients.

**Clinical variables**	**TCGA cohort**	**Clinical variables**	**GSE65904 cohort**
**Survival status**		**Survival status**	
Dead	224	Dead	102
Survived	239	Survived	108
Unknown		Unknown	4
Median age (year)	57.84	Median age (year)	62.35
**Sex**		**Sex**	
Female	174	Female	89
Male	289	Male	124
Unknown		Unknown	1
**T stage**		**Tumor stage**	
0	23	General	23
1	42	In-transit	15
2	77	Local	11
3	91	Primary	16
4	150	Regional	139
Unknown	80	Unknown	10
**N stage**		**Tissue**	
0	227	Cutaneous	22
1	75	Lymph node	130
2	50	Subcutaneous	33
3	57	Visceral	10
Unknown	54	Unknown	19
**M stage**			
0	411		
1	24		
Unknown	28		
**AJCC stage**			
1	78		
2	136		
3	174		
4	23		
Unknown	52		
**Metastatic status**			
Metastatic	361		
Primary	99		
Others	3		

### Kyoto Encyclopedia of Genes and Genomes (KEGG) pathway enrichment analysis and Gene Ontology (GO)

KEGG enrichment analysis and GO of OS-associated differentially expressed genes (DEGs) were performed to assess their biological functions. The Database for Annotation, Visualization, and Integrated Discovery 6.8 (Huang, Sherman & Lempicki, 2009) was used to perform the analyses.

### Establishing protein-protein interaction (PPI) network and screening for important modules

PPI information from OS-associated DEGs was acquired from the STRING platform (http://www.string-db.org/) (Szklarczyk et al., 2019). The PPI network was developed using Cytoscape 3.7.0. The virtual modules and hub genes in the PPI network with a Molecular Complex Detection (MCODE) score and node count >5 (*p* < 0.05) were selected using the MCODE plug-in (Bader & Hogue, 2003).

### Construction of a prognostic risk model

The univariate Cox regression of hub genes found in the PPI network was performed using the ‘survival’ R package. Subsequently, multivariate Cox regression was used to further analyze the above OS-related genes and select genes to build the prognostic risk model. We calculated the risk value of patients with SKCM based on the expression and coefficient values of 16 OS genes and classified them into high- and low-risk queues through the median risk score. The risk score was determined according to the equation below:

\begin{eqnarray*}\text{Risk score}=\Sigma \mathrm{expgenei}\times \beta i, \end{eqnarray*}

where expgenei is the expression level of 16 OS-associated genes and *β* is the coefficient value for the gene. Receiver operating characteristic (ROC) curves were generated using the ‘timeROC’ and ‘survivalROC’ packages for R and were applied to assess the accuracy of our risk model to predict the overall survival rate of SKCM patients. Separate nomograms were constructed based on clinical characteristics and the 16 OS genes. The calibration chart was used to detect the predictive power of the above nomograms (one based on clinical characteristics and one based on the 16 OS genes) for the overall survival time of SKCM patients. To validate the prognostic performance of the constructed risk model, the analyses described above were conducted using data from the GSE65904 cohort.

### Validation of expression levels and prognostic values of hub genes

After clarifying the translational expression level of hub genes in the TCGA cohort, we verified the differential expression of 16 OS genes between SKCM and normal skin tissues using data from the Human Protein Atlas (HPA) (Thul et al., 2017). In the TCGA cohort, a survival analysis of 16 OS genes was performed using the Kaplan–Meier (KM) approach to ascertain whether they were correlated with the prognosis of SKCM patients.

## Results

### Identification of OS-associated DEGs

**Figure 1:**
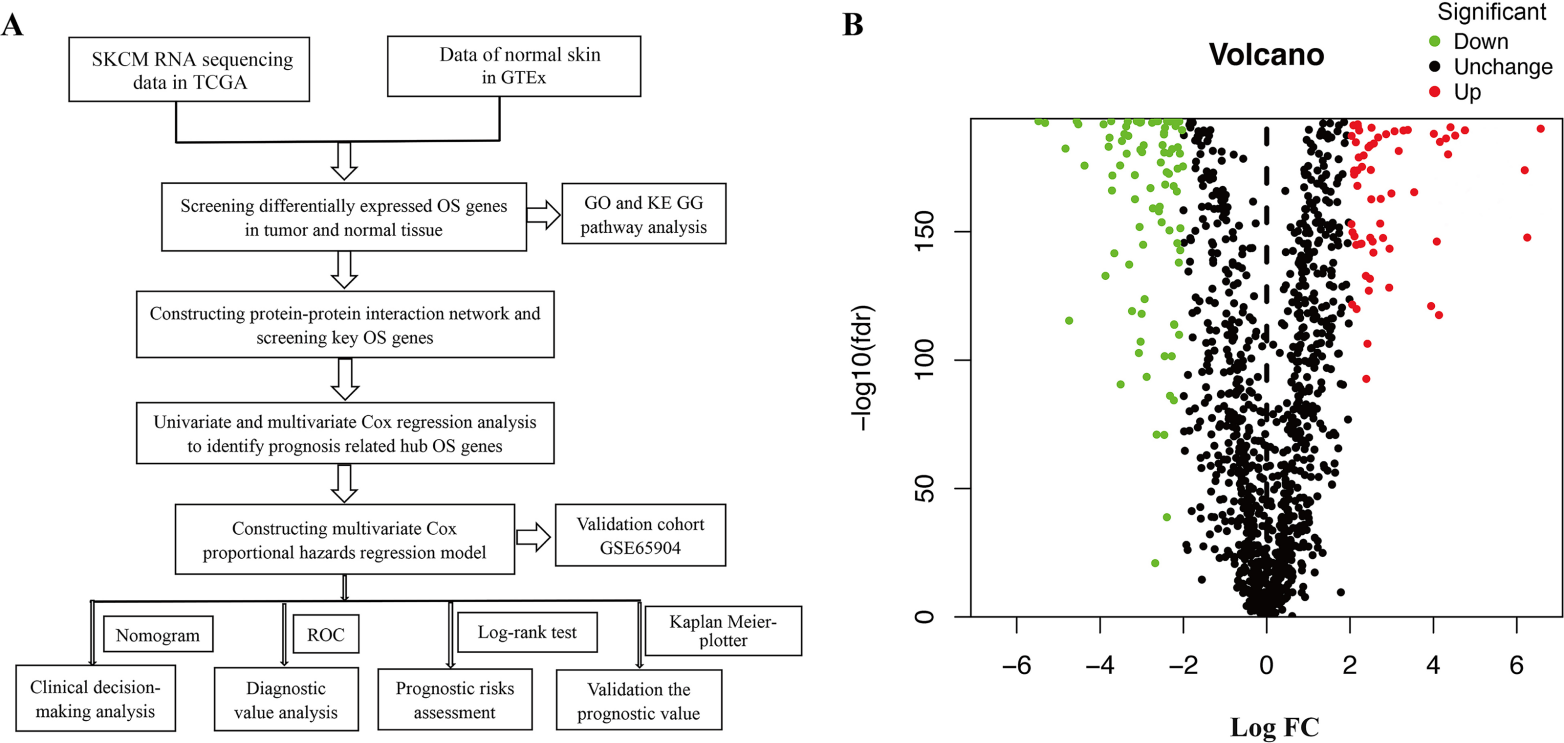
(A) Flowchart describing the schematic overview of the study design. (B) Volcano plot of OS-associated DEGs in TCGA-SKCM cohort.

Figure 1A shows the workflow of the study. In total, 1399 OS genes were selected to study their differential expression in SKCM and healthy tissues. Of these, 156 were identified as OS-associated DEGs (63 upregulated and 93 downregulated genes) in SKCM (Fig. 1B).

### Functional enrichment analysis of OS-associated DEGs

**Figure 2:**
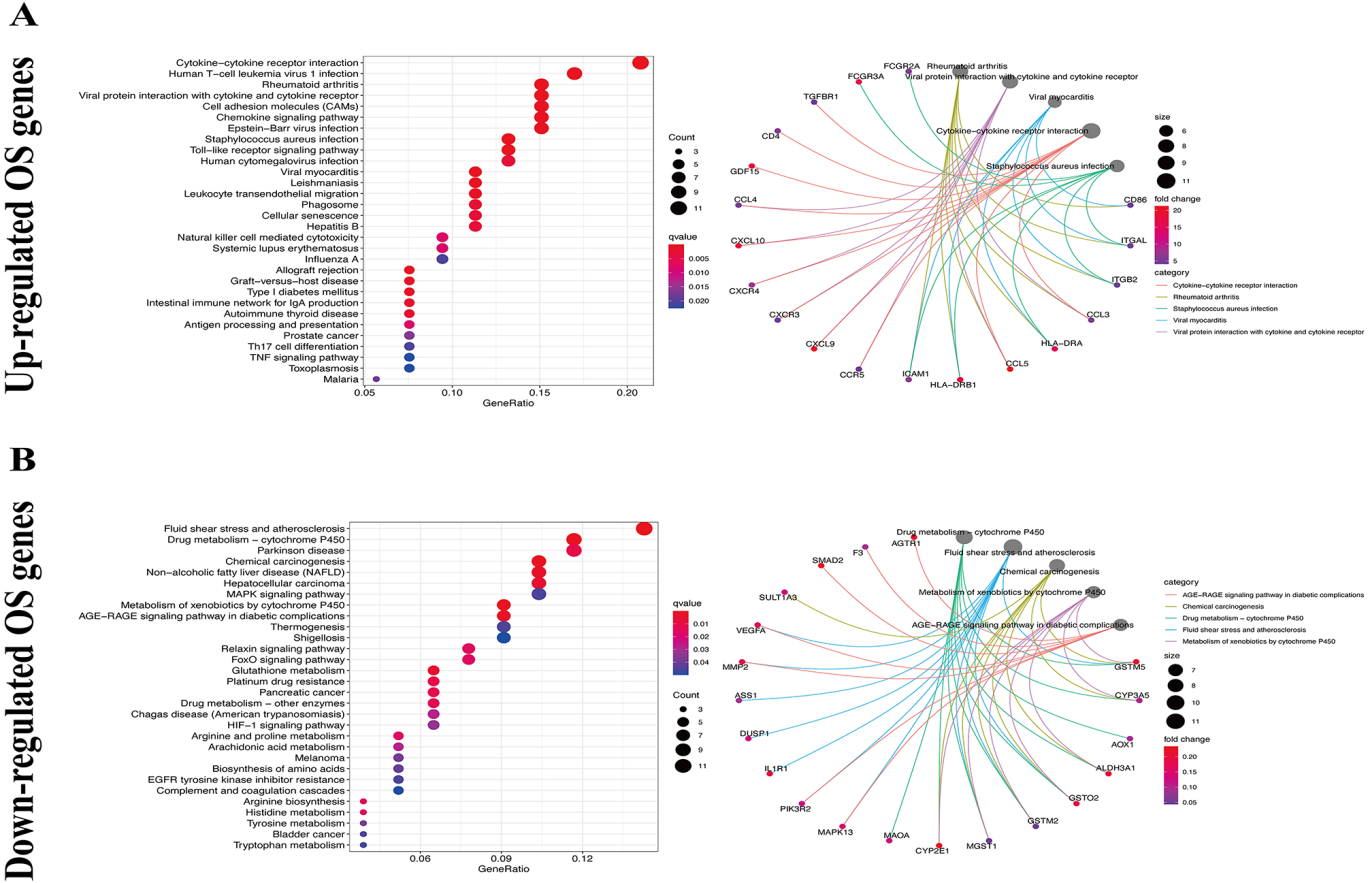
KEGG enrichment analysis of OS-associated DEGs. (A) Top 30 classes of KEGG enrichment terms about up-regulated DEGs. (B) Top 30 classes of KEGG enrichment terms about down-regulated DEGs.

**Figure 3:**
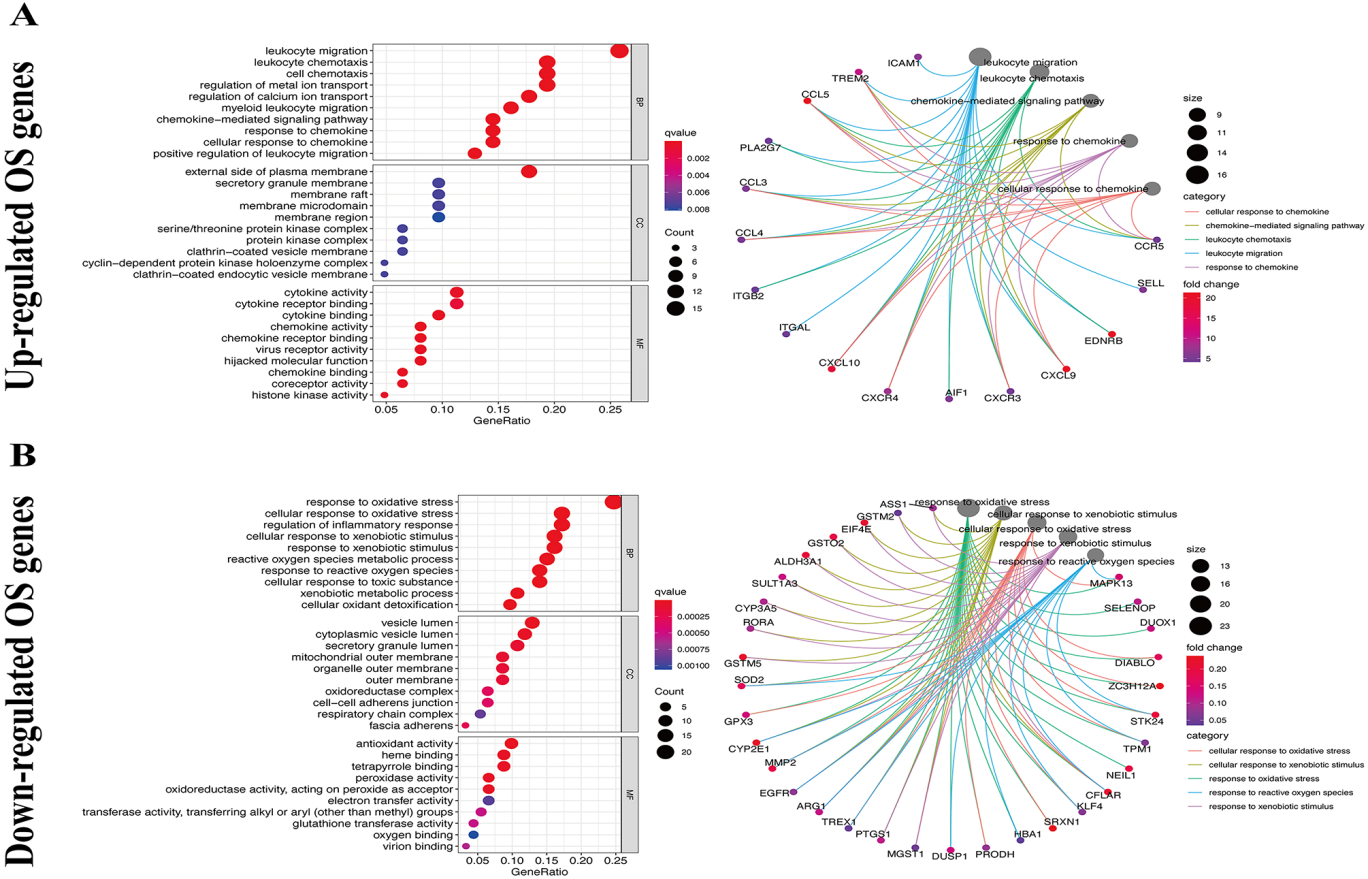
GO enrichment analysis of OS-associated DEGs. (A) Top 10 classes of GO enrichment terms about up-regulated DEGs in biological process (BP), cellular component (CC), and molecular function (MF). (B) Top 10 classes of GO enrichment terms about down-regulated DEGs in BP, CC, and MF.

We used the KEGG pathway analysis to analyze the DEGs and found that upregulated genes were mostly correlated with cytokin-cytokin receptor interaction and hunman T-cell leukemia virus 1 infection (Fig. 2A), while downregulated genes were predominantly linked with fluid shear stress and atherosclerosis (Fig. 2B). GO analysis was also performed to further explore the DEGs. As a result, with regard to biological processes, upregulated DEGs were significantly augmented in leukocyte migration and leukocyte chemotaxis (Fig. 3A), whereas DEGs that were downregulated were mainly augmented in response to OS and cellulare response to OS (Fig. 3B). With regard to cell location, upregulated genes were mainly augmented on the external side of plasma membrane and secretory granule membrance (Fig. 3A). Downregulated genes were enriched in the vesicle lumen and cytoplasmic vesicle lumen (Fig. 3B). Finally, it was evident that upregulated OS genes were concentrated in cytokine activity and cytokine receptor binding (Fig. 3A), while downregulated OS genes were mostly implicated in antioxidant activity and heme binding (Fig. 3B).

### Creation of a PPI network for OS-associated DEGs and screening of key modules

**Figure 4:**
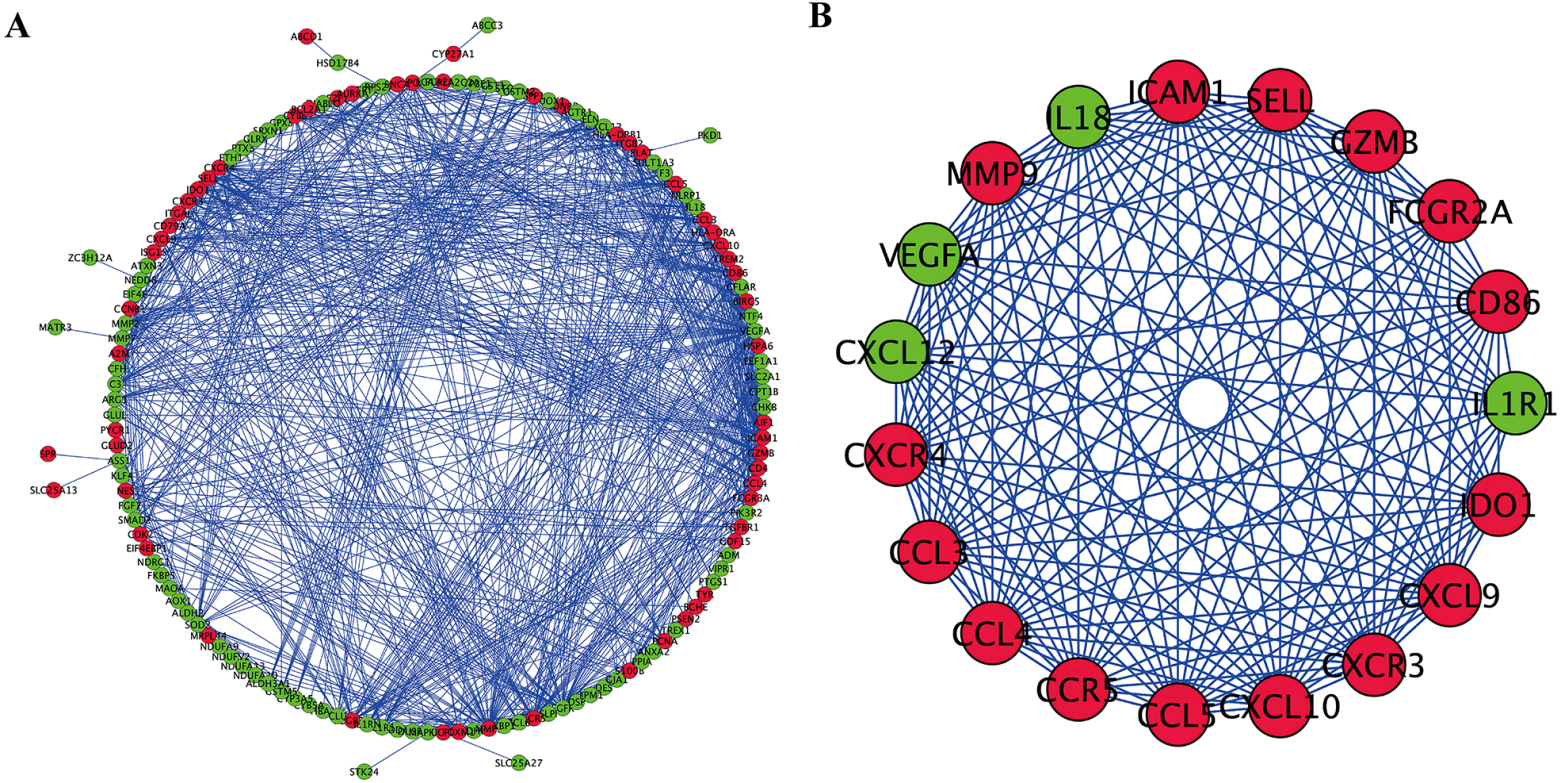
PPI network and modules screening. (A) PPI network of OS-associated DEGs. (B) Critical module from PPI network. Green circles represent down-regulated genes, and red circles represent up-regulated genes.

To further explore the inner relationship of OS-associated DEGs, a PPI network was established with 144 nodes and 834 edges (Fig. 4A). The most meaningful module with 19 nodes and 158 edges was subsequently identified (Fig. 4B). OS-related genes within the key module were primarily involved in chemokine-mediated signaling transduction, leukocyte migration, response to chemokine, and chemokine signaling pathway.

### Screening of hub genes and construction of a prognostic risk model

**Figure 5:**
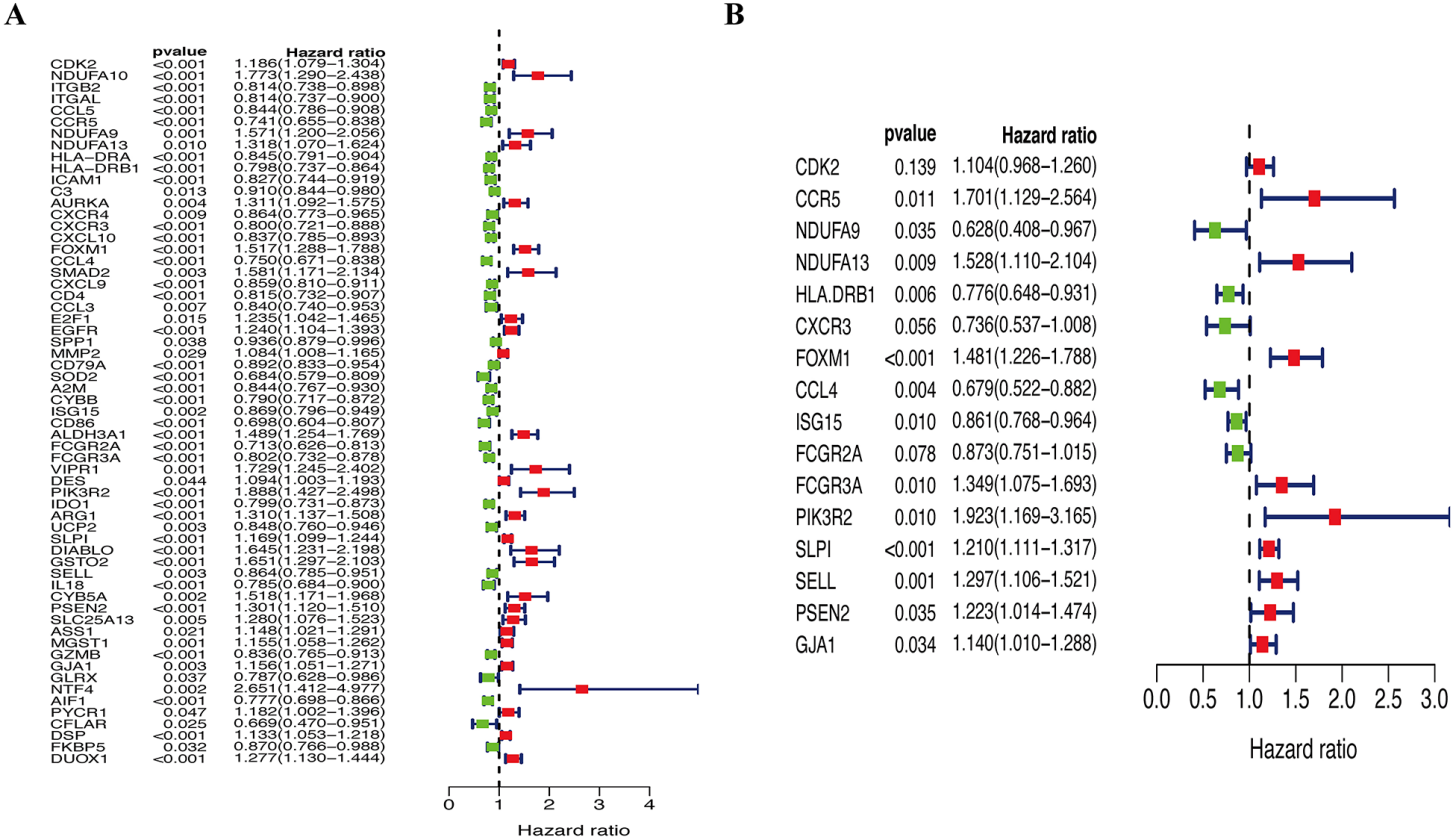
Identification of hub OS genes in TCGA cohort. (A) Univariate Cox regression analysis for identification prognosis-associated OS genes. (B) Multivariate Cox proportional hazards regression model was constructed based on the identified prognostic-related OS genes.

A total of 144 OS-associated DEGs were identified from the PPI network. After univariate Cox regressions, 61 OS genes were identified as genes of prognostic value in SKCM patients (Fig. 5A). The multivariate Cox regression model helped to select 16 hub genes (CDK2, CCR5, NDUFA9, NDUFA13, HLA.DRB1, CXCR3, FOXM1, CCL4, ISG15, FCGR2A, FCGR3A, PIK3R2, SLPI, SELL, PSEN2, and GJA1) that were used to calculate the prognostic risk model (Fig. 5B, Table 2).

**Figure 6:**
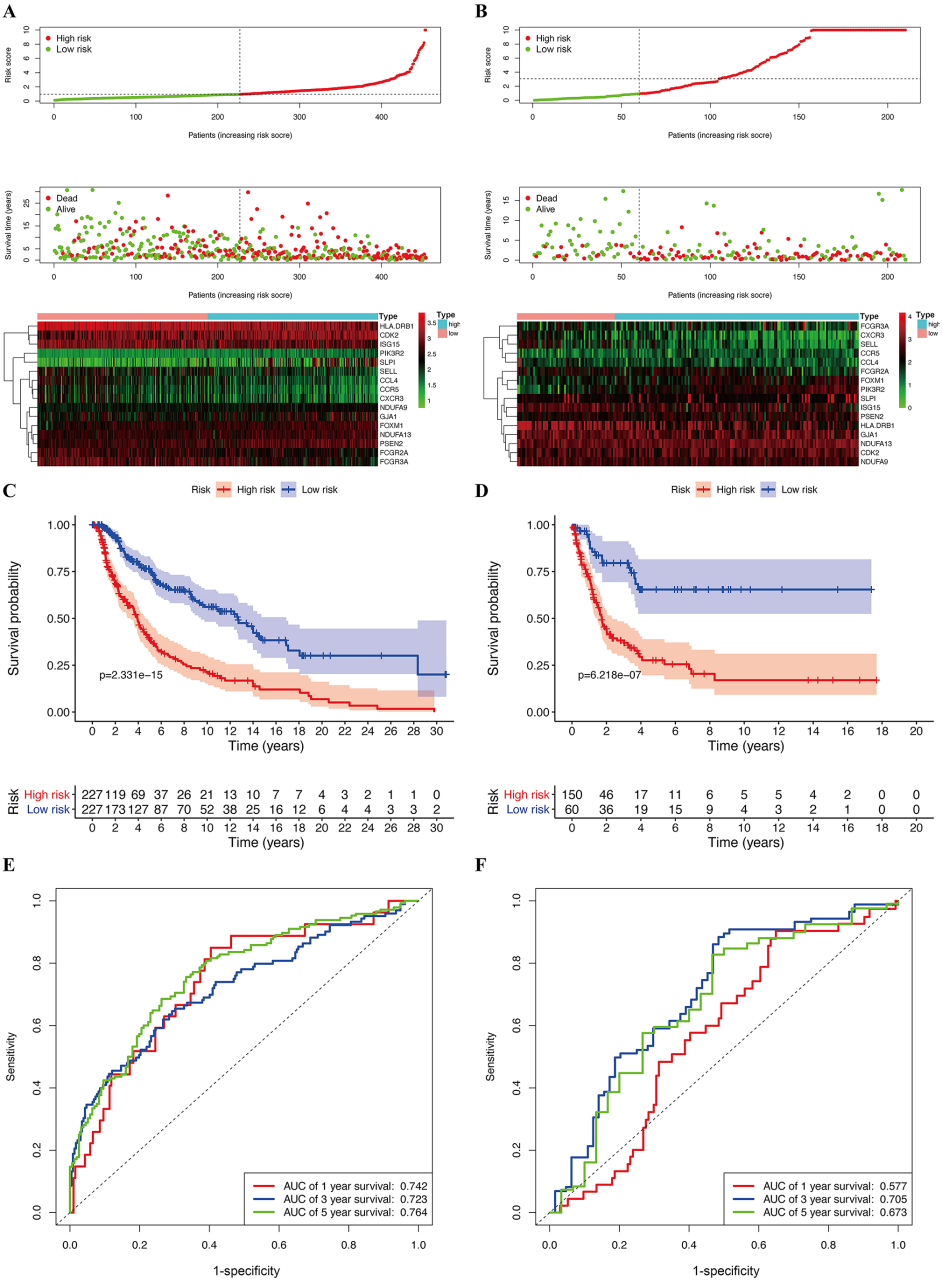
Construction of prognostic model in the TCGA and GSE65904 cohort. (A) Risk score distribution, survival status, and expression heat map of TCGA cohort. (B) Risk score distribution, survival status, and expression heat map of GSE cohort. (C) Survival curve of TCGA cohort. (D) Survival curve of GSE cohort. (E) ROC curves for forecasting overall survival in TCGA cohort. (F) ROC curves for forecasting overall survival in GSE cohort.

**Table 2:** Sixteen prognosis-associated hub OS genes identified by multivariate Cox regression analysis.

**Symbol**	**coef**	**HR**	**Lower 95% CI**	**High 95% CI**	***P*-value**
CDK2	0.0993	1.1044	0.9682	1.2597	0.1393
CCR5	0.5314	1.7014	1.1290	2.5638	0.0111
NDUFA9	−0.4646	0.6284	0.4083	0.9672	0.0347
NDUFA13	0.4240	1.5280	1.1096	2.1042	0.0094
HLA.DRB1	−0.2531	0.7764	0.6477	0.9307	0.0062
CXCR3	−0.3069	0.7358	0.5371	1.0080	0.0561
FOXM1	0.3925	1.4807	1.2259	1.7883	0.0000
CCL4	−0.3873	0.6789	0.5224	0.8823	0.0038
ISG15	−0.1498	0.8609	0.7684	0.9645	0.0098
FCGR2A	−0.1357	0.8731	0.7509	1.0152	0.0777
FCGR3A	0.2993	1.3489	1.0747	1.6931	0.0098
PIK3R2	0.6541	1.9234	1.1688	3.1653	0.0101
SLPI	0.1903	1.2096	1.1106	1.3174	0.0000
SELL	0.2599	1.2968	1.1058	1.5208	0.0014
PSEN2	0.2011	1.2228	1.0143	1.4741	0.0349
GJA1	0.1313	1.1403	1.0096	1.2879	0.0345

### Validation of the prognostic value of the risk model

**Figure 7:**
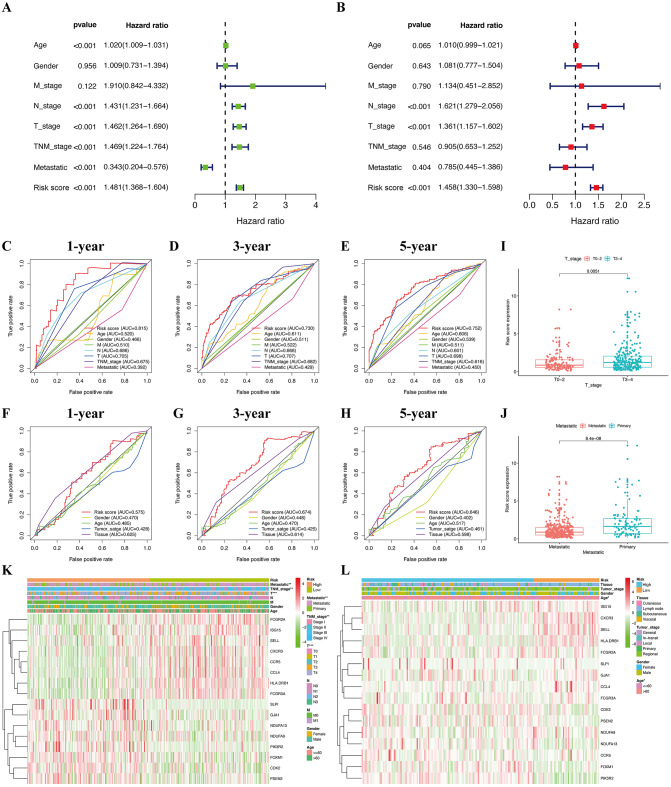
Efficacy evaluation of constructed prognostic model. Univariate (A) and multivariate (B) Cox regression analysis of the clinicopathological features in TCGA cohort. ROC curves for forecasting overall survival in TCGA (C-E) and GSE65904 (F-H) cohort. (I) The relationship between the risk scores and T stage in TCGA cohort. (J) The relationship between the risk scores and metastatic ability in TCGA cohort.The heatmap shows the distribution of clinicopathological features and OS genes expression in TCGA (K) and GSE65904 (L) cohort.

The median risk score of the prognostic risk model was used to separate SKCM patients in the TCGA and GSE65904 cohorts into high- and low-risk queues (Figs. 6A, 6B). The survival time of SKCM patients in the high-risk group was significantly lower when compared to the low-risk group (Figs. 6C, 6D). A ROC curve was constructed to validate the accuracy of our risk model. It indicated that our risk model had a moderate predictive effect using data from the TCGA cohort (area under the ROC curve [AUC] of 1-year survival = 0.742; 3-year survival = 0.723; and 5-year survival = 0.764) (Fig. 6E). Similarly, both the prognostic effect and accuracy were validated in the GSE65904 cohort, in which the AUC of 3-year survival was 0.705 (Fig. 6F). The univariate and multivariate Cox regressions of different clinical characteristics of SKCM patients in the TCGA cohort showed that the gene-based risk score was a robust prognostic parameter for SKCM patients (Figs. 7A, 7B). When compared with other clinical features in the TCGA and GSE65904 cohort, our prognostic risk model showed a better prognostic performance in all AUCs (Figs. 7C–7H), revealing that our gene-based prognostic risk model displayed moderate specificity and sensitivity with regard to predicting SKCM prognosis. The correlation analysis between clinical parameters and risk score indicated that SKCM patients in the T3 and T4 stages or those with primary cancer had a higher risk score. (Figs. 7I, 7J). A heatmap was drawn to show the correlation between the levels of 16 OS genes in the TCGA and GSE65904 cohorts against different clinical parameters, including high- and low-risk groups, TNM stage, age, and gender (Fig. 7K, 7L).

**Figure 8:**
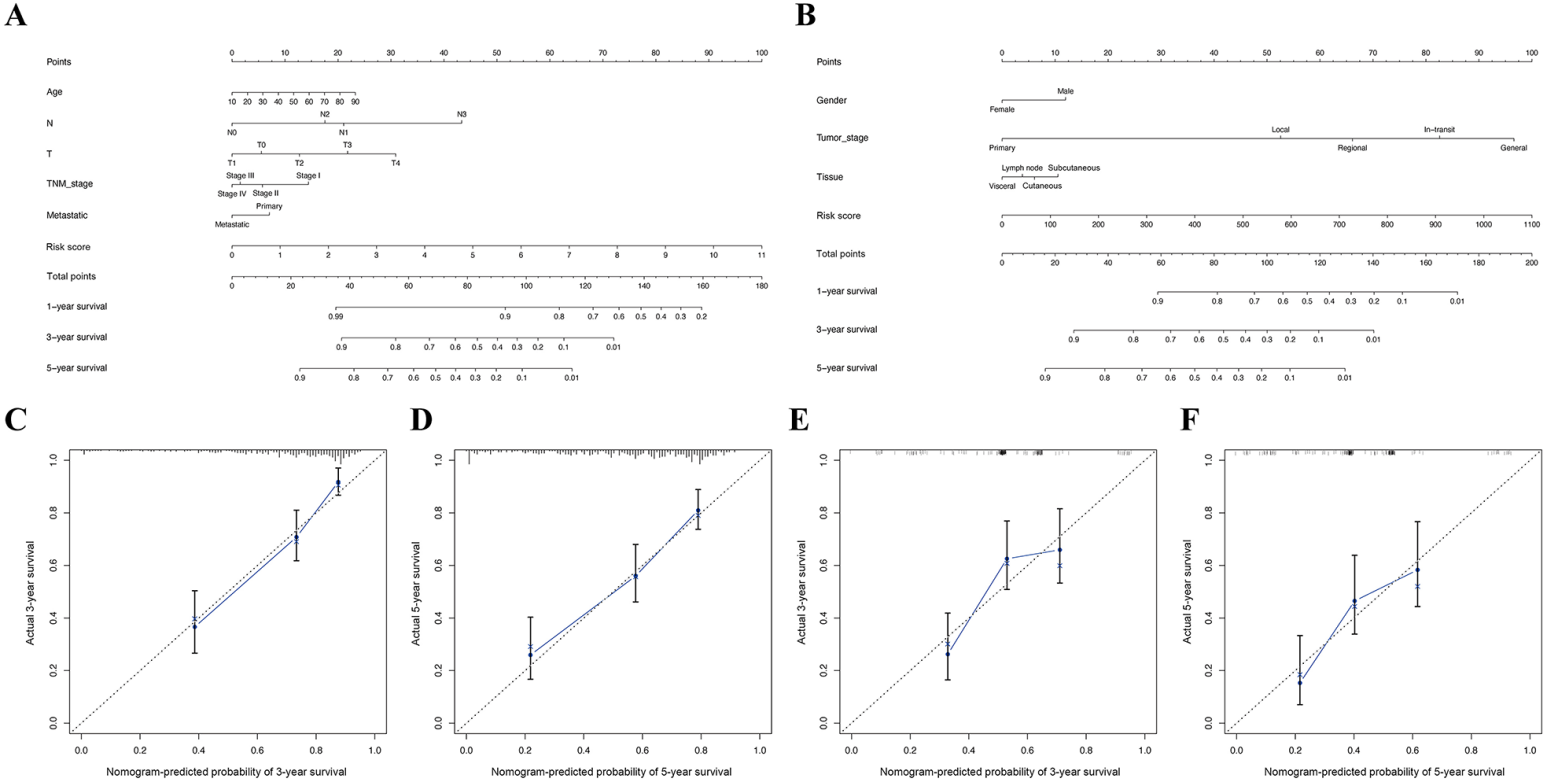
Construction of nomogram based on the risk score and other clinical factors. Nomograms for predicting SKCM 1-, 3-, and 5-year overall survival in TCGA (A) and GSE65904 (B) cohort. (C-D) The calibration plot of the nomogram in TCGA cohort. (E-F) The calibration plot of the nomogram in GSE65904 cohort.

Furthermore, in the TCGA and GSE65904 cohorts, the nomograms of our risk score and different clinical parameters were constructed to predict the overall prognosis of SKCM patients (Fig. 8A, 7B). In parallel, the calibration diagram evidenced that the above nomograms had a good predictive effect on the clinical outcome of SKCM patients (Figs. 8C–8F).

### Validating the prognostic value and expression levels of hub genes

In SKCM samples, the expression levels of CDK2, CCR5, HLA-DRB1, CXCR3, FOXM1, CCL4, ISG15, FCGR2A, FCGR3A, SELL, and PSEN2 were considerably increased, while the levels of NDUFA9, NDUFA13, PIK3R2, SLPI, and GJA1 were markedly decreased when compared with that in healthy samples (File S1A). These results were validated by immunohistochemistry analysis from the HPA database (File S1B).

**Figure 9:**
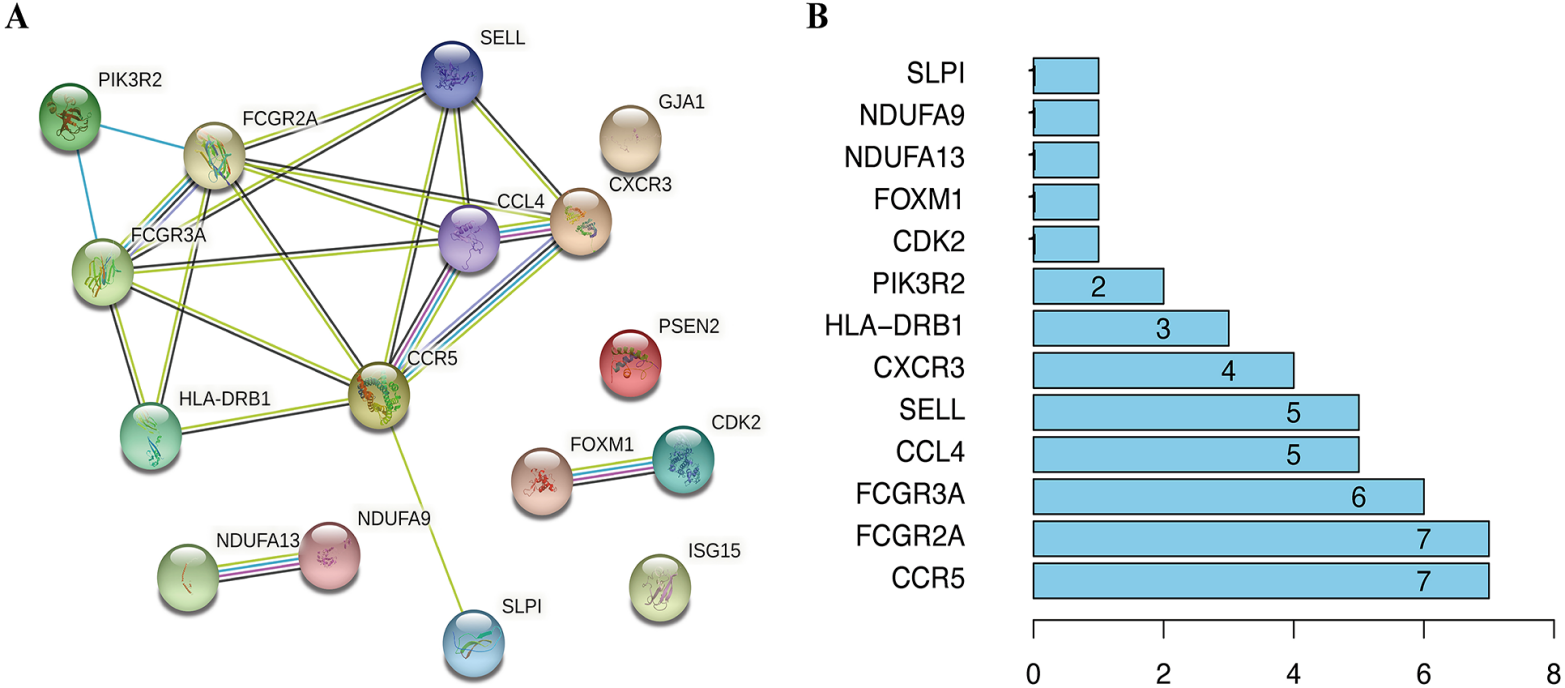
The interactions between identified hub DEGs. (A) The PPI network of 16 prognosis-associated OS genes based on STRING database. (B) Spearman correlation analysis of 16 OS genes.

To visualize the interactions between hub DEGs, we constructed a PPI network using the STRING database online tool (Fig. 9A). The genes FCGR2A and CCR5 showed the highest interacting degrees among the hub genes (Fig. 9B). The KM method showed that a high expression level of HLA-DRB1, CXCR3, CCL4, ISG15, FCGR2A, FCGR3A, SELL, and CCR5 were correlated with a significant increase in the overall survival rate in SKCM, whereas a high expression level of PSEN2, CDK2, FOXM1, GJA1, NDUFA9, NDUFA13, PIK3R2, and SLPI were correlated with a significant decrease of the overall survival rate (File S2). Moreover, as shown in File S3, the genes SELL, PSNE2, FOXM1, CDK2, and HDUFA9 were all significantly related to with SKCM ages, while genes HDUFA13 and CCL4 were significantly connected the ganders of SKCM patients. For the TCGA and GSE65904 cohorts, we also constructed the nomograms related to the 16 OS genes to predict the 1-, 3-, and 5-year survival probability of SKCM patients (File S4). The calibration of the nomograms associated with the 16 OS genes presented good consistency between the predicted and observed outcomes.

## Discussion

The incidence of SKCM has increased over the past 50 years, and it ranks 19th among the most common malignant tumors worldwide (Holmes, 2014). Currently, the management of SKCM is through surgical resection, although it does not sufficiently improve the overall survival rate (Swetter et al., 2019). OS is known to be involved in the occurrence and development of several tumors (Kruk & Aboul-Enein, 2017), however the prognostic value of OS genes on tumor survival remains unclear. Here, we sought to identify molecular biomarkers to predict the prognosis of SKCM and provide a rationale for decisions regarding treatment. Therefore, we analyzed the differential expression of OS-related genes between SKCM and normal samples, obtaining 156 DEGs (63 upregulated and 93 downregulated genes). GO enrichment analysis indicated that DEGs were mainly involved in OS, chemokine, and ROS-associated functions, whereas KEGG pathway analysis suggested that they could have a significant impact on the initiation and growth of certain tumors, such as prostate cancer, hepatocellular carcinoma, pancreatic cancer, bladder cancer, and especially melanoma.

A PPI network was built to analyze the interactions between OS-associated DEGs and identify a key module. Furthermore, univariate and multivariate Cox regressions revealed 16 hub genes, including HLA-DRB1, CXCR3, CCL4, ISG15, FCGR2A, FCGR3A, SELL, CCR5, PSEN2, CDK2, FOXM1, GJA1, NDUFA9, NDUFA13, PIK3R2, and SLPI. Interestingly, these genes were found to have several cancer-related roles: CXCR3 can interact with LRP1 leading to ligand-induced conformational changes on the cell membrane, which results in increased tumor cell migration (Boyé et al., 2017); ISG15 is highly expressed in hepatocellular carcinoma tissues and interacts with XIAP to drive proliferation and metastasis (Li et al., 2014; Tong et al., 2020); CCR5 is positively associated with the size of the primary tumor (Suarez-Carmona et al., 2019), whereas its overexpression significantly promotes leukocyte accumulation, angiogenesis, and tumor progression in oral squamous cell carcinoma (Da Silva et al., 2017); NDUFA9 is related to colitis-associated cancer and may be connected with the activation of the LKB1/AMPK pathway in colorectal epithelial cells (Wang, Cui & Qu, 2019); PIK3R2 is closely related to liver cancer prognosis, since its overexpression significantly increases the probability of liver cancer metastasis and angiogenesis (Du et al., 2014); and SLPI provides a local immune response to human papillomavirus infection in the cervical mucosa (Sahin et al., 2018), while its modulation significantly inhibits the expression of apoptosis-associated genes, promoting the proliferation and metastasis of gastric cancer (Du et al., 2017; Sahin et al., 2018). Although the modulation effects of these genes had been explored in various tumors, few studies have systematically analyzed their specific prognostic values in SKCM.

In the present study, the survival analysis results obtained through the KM method showed that the expression levels of 16 OS-related genes were associated with SKCM patients’ survival. Elevated expression of PSEN2, CDK2, FOXM1, GJA1, NDUFA9, NDUFA13, PIK3R2, and SLPI was associated with a lower survival rate, indicating that these genes may be oncogenes. Conversely, overexpression of HLA-DRB1, CXCR3, CCL4, ISG15, FCGR2A, FCGR3A, SELL, and CCR5 was associated with a significantly higher survival rate, revealing their vital role in inhibiting the progression of cancer.

The ROC curves and survival analyses confirmed the advanced biological implications of our model to predict the outcomes of SKCM patients. In addition, it showed an improved predictive accuracy when compared to other clinical parameters. Cox regressions evidenced that our risk score was an independent prognostic parameter for SKCM patients. Nomograms constructed based on the gene expression levels and risk signature ascertained the credibility of our risk model to estimate the overall survival time of SKCM patients. Given the fundamental role of OS in SKCM metastasis and progression (Obrador et al., 2019), we also detected the relationship between the clinical factors and calculated risk score. The results suggested that our risk model was able to estimate the metastasis and T stage of patients with SKCM, highlighting its high correlation with cancer prognosis and progression.

Nonetheless, there are some limitations in this study. First, this study was designed as a retrospective analysis; more prospective research should be performed to verify our results. Second, our results lack in vitro or in vivo exploration to confirm the reliability of our mechanism analysis. Therefore, we need to conduct several further experiments to prove the mechanistic connections between these genes and SKCM.

## Conclusion

In conclusion, we systematically studied prognosis-associated OS genes for SKCM using a series of bioinformatics techniques and identified 16 hub genes that were correlated with overall survival rate. We also successfully developed and validated a prognostic risk model for melanoma using OS genes. Overall, this result may help to study the progression and metastasis of SKCM more thoroughly and provide a deeper understanding of the mechanisms involved in these processes.

##  Supplemental Information

10.7717/peerj.11258/supp-1 Supplemental Information 1 The expression of prognosis-associated OS genes in SKCM patients (A) The Violin plot reveals the transcription expression of hub OS genes in TCGA cohort. (B) HPA database verifies the protein expression level of hub OS genes in SKCM.

10.7717/peerj.11258/supp-2 Supplemental Information 2 Validation the prognostic value of 16 prognosis-associated OS genes in TCGA cohort by Kaplan-Meier analysis

10.7717/peerj.11258/supp-3 Supplemental Information 3 The relationship between the gene expression level and tumor ages or genders in TCGA cohort

10.7717/peerj.11258/supp-4 Supplemental Information 4 Construction of nomogram based on the expression of 16 OS genes The nomogram (A) and calibration plot (C) of 16 OS genes in TCGA cohort. The nomogram (B) and calibration plot (D) of 16 OS genes in GSE65904 cohort.

## Additional Information and Declarations

### Data Availability

The following information was supplied regarding data availability: All gene expression profiles are available from the TCGA (https://portal.gdc.cancer.gov; search term: cases.primary_site in [“skin”] and cases.project.program.name in [“TCGA”] and cases.project.project_id in [“TCGA-SKCM”] and files.analysis.workflow_type in [“HTSeq-FPKM”] and files.data_category in [“transcriptome profiling”] and files.data_type in [“Gene Expression Quantification”] and files.experimental_strategy in [“RNA-Seq”]), UCSC Xena, (https://xenabrowser.net/datapages/?dataset=TCGA-SKCM.htseq_fpkm.tsv&host=https%3A%2F%2Fgdc.xenahubs.net&removeHub=https%3A%2F%2Fxena.treehouse.gi.ucsc.edu%3A443), GTEx (https://xenabrowser.net/datapages/?dataset=gtex_RSEM_gene_fpkm&host=https%3A%2F%2Ftoil.xenahubs.net&removeHub=https%3A%2F%2Fxena.treehouse.gi.ucsc.edu%3A443) and GSE65904.
