# Supplementary figures and images for: Development and validation of an oxidative stress—associated prognostic risk model for melanoma
Source: PeerJ. 2021 Apr 20;9:e11258. doi: 10.7717/peerj.11258 (PMC8063880; doi:10.7717/peerj.11258)

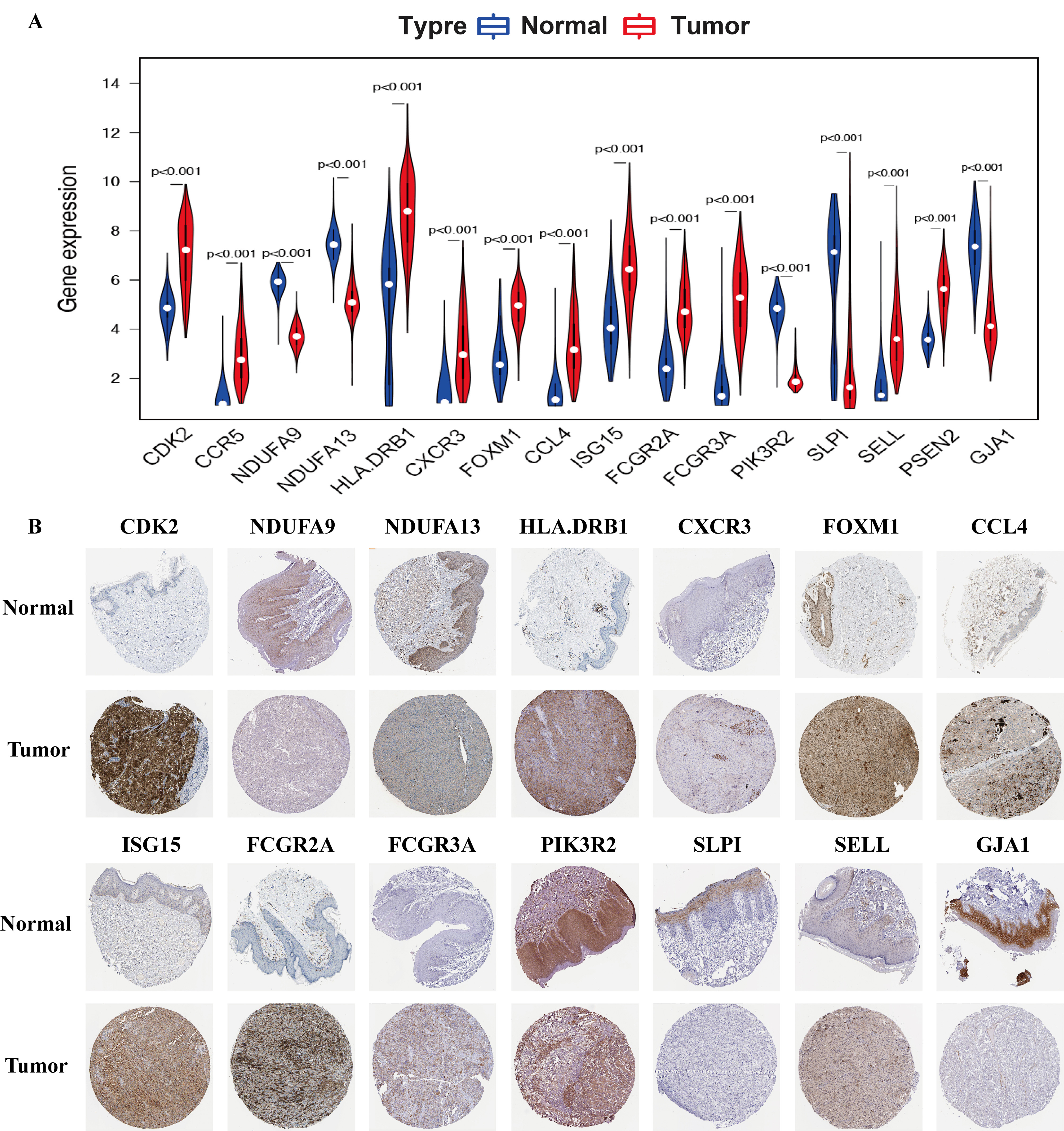

Supplement: Supplemental Information 1 — (A) The Violin plot reveals the transcription expression of hub OS genes in TCGA cohort. (B) HPA database verifies the protein expression level of hub OS genes in SKCM. [file peerj-09-11258-s001.jpg]

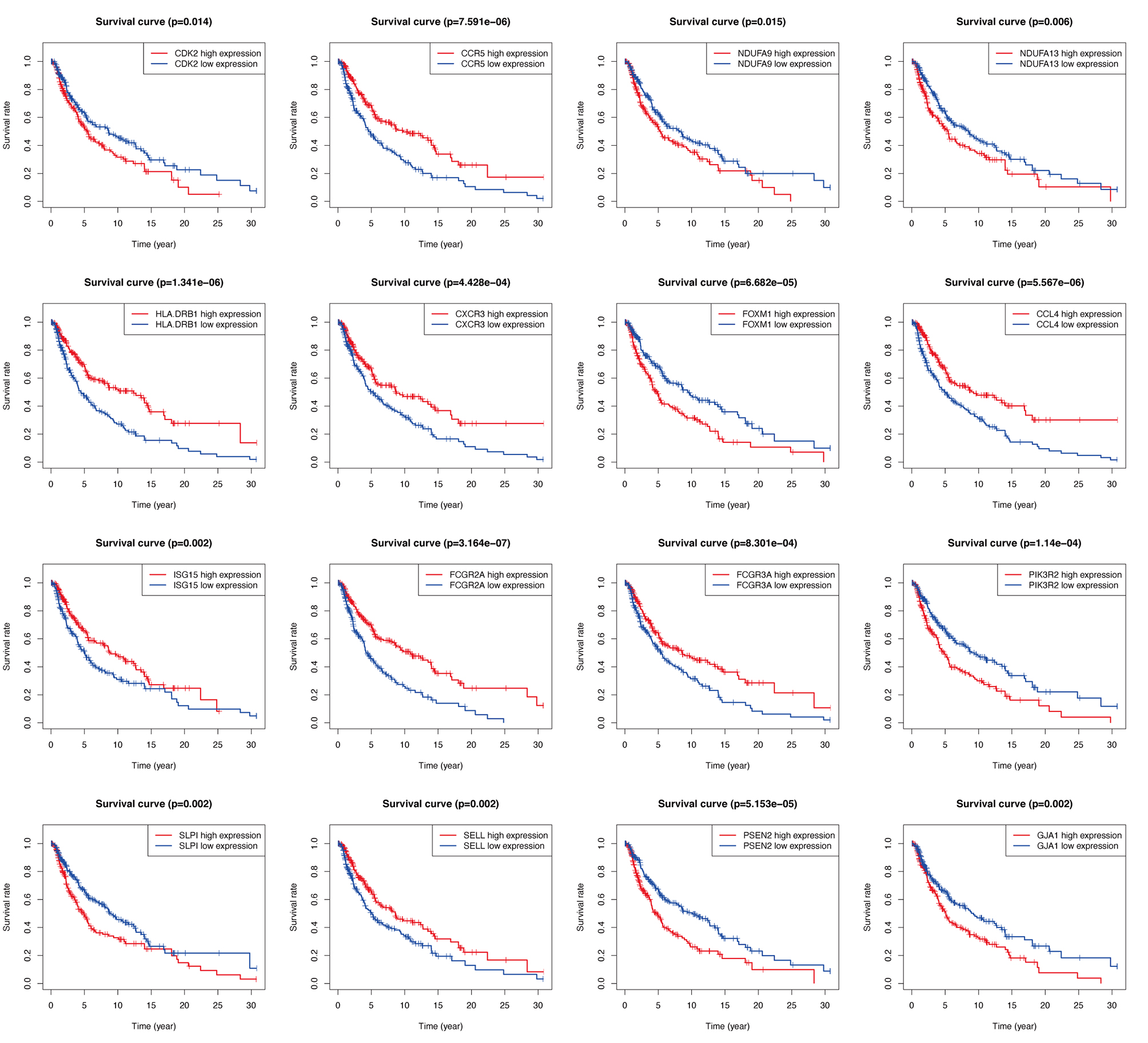

Supplement: Supplemental Information 2 [file peerj-09-11258-s002.jpg]

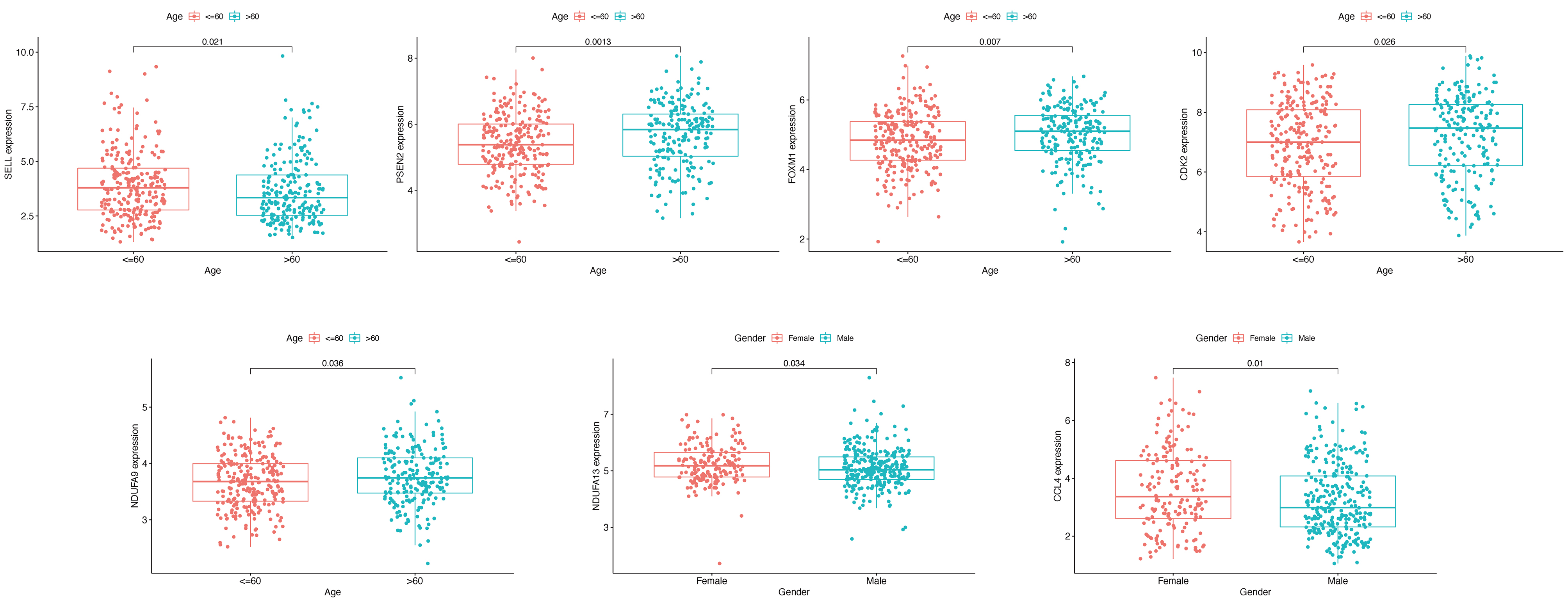

Supplement: Supplemental Information 3 [file peerj-09-11258-s003.jpg]

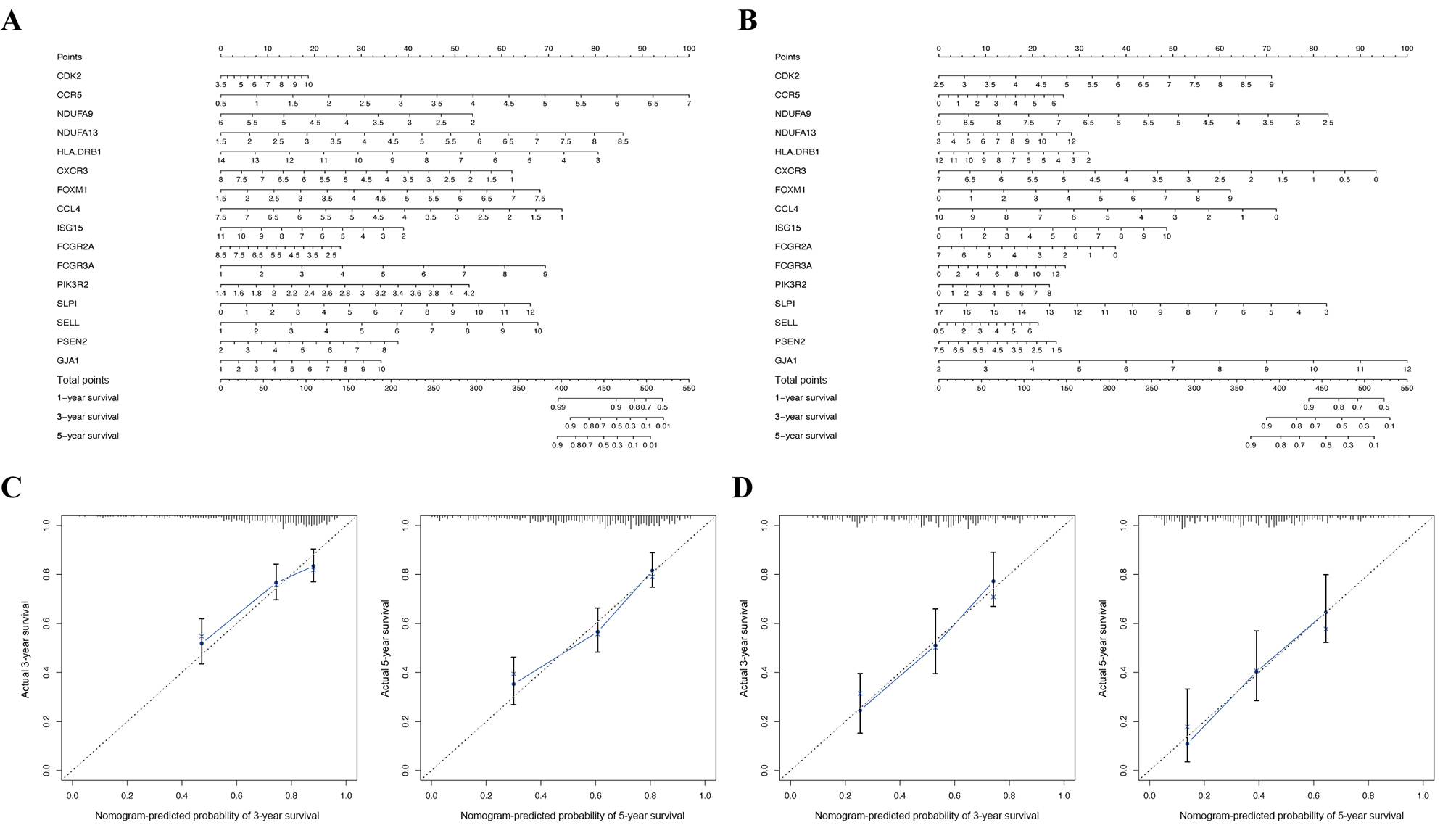

Supplement: Supplemental Information 4 — The nomogram (A) and calibration plot (C) of 16 OS genes in TCGA cohort. The nomogram (B) and calibration plot (D) of 16 OS genes in GSE65904 cohort. [file peerj-09-11258-s004.jpg]
